# The Benefits of the Citrus Flavonoid Diosmin on Human Retinal Pigment Epithelial Cells under High-Glucose Conditions

**DOI:** 10.3390/molecules22122251

**Published:** 2017-12-18

**Authors:** Wayne Young Liu, Shorong-Shii Liou, Tang-Yao Hong, I-Min Liu

**Affiliations:** 1 Department of Urology, Jen-Ai Hospital, Taichung City 41625, Taiwan; waynedoctor@gmail.com; 2 Center for Basic Medical Science, College of Health Science, Central Taiwan University of Science and Technology, Taichung City 40601, Taiwan; 3 Department of Pharmacy and Master Program, College of Pharmacy and Health Care, Tajen University, Pingtung County 90741, Taiwan; ssliou@tajen.edu.tw; 4 Department of Biotechnology, College of Pharmacy and Health Care, Tajen University, Pingtung County 90741, Taiwan; tyhong@tajen.edu.tw

**Keywords:** diosmin, human retinal pigment epithelial cells, high glucose, diabetic retinopathy, ROS, MAPK

## Abstract

We investigate diosmin for its effect on the ARPE-19 human retinal pigment epithelial cells exposed to high glucose, a model of diabetic retinopathy (DR). After incubation for 4 days with a normal (5 mmol/L) concentration of D-glucose, ARPE-19 cells were exposed separately to normal or high concentrations of D-glucose (30 mmol/L) with or without diosmin at different concentrations (0.1, 1, 10 μg/mL) for another 48 h. Next, we assessed cell viability, reactive oxygen species (ROS) generation and antioxidant enzyme activities. In order to examine the underlying molecular mechanisms, we meanwhile analyzed the expressions of Bax, Bcl-2, total and phosphorylated JNK and p38 mitogen-activated protein kinase (MAPK). Diosmin dose dependently enhanced cell viability following high glucose treatment in ARPE-19 cells. The activities of superoxide dismutase and glutathione peroxidase, as well as the levels of reduced glutathione were decreased, while it was observed that levels of ROS in high glucose cultured ARPE-19 cells increased. High glucose also disturbed Bax and Bcl-2 expression, interrupted Bcl-2/Bax balance, and triggered subsequent cytochrome c release into the cytosol and activation of caspase-3. These detrimental effects were ameliorated dose dependently by diosmin. Furthermore, diosmin could abrogate high glucose-induced apoptosis as well as JNK and P38 MAPK phosphorylation in ARPE-19 cells. Our results suggest that treatment ARPE-19 cells with diosmin halts hyperglycemia-mediated oxidative damage and thus this compound may be a candidate for preventing the visual impairment caused by DR.

## 1. Introduction

Diabetic retinopathy (DR) is a prevalent retinal disease and a leading cause of blindness in diabetic patients worldwide. The basis for the loss of vision associated with this disease has much to do with the oxidative damage inflicted on retinal pigment epithelial (RPE) cells by high glucose [1]. The RPE is a highly specialized retinal cell layer, which serves as a crucial role in the survival and function of photoreceptors in the retina [2]. When reacting to injury or alterations in the hyperglycemic environment, RPE cells migrate and proliferate, leading to a breakdown in adhesion between the RPE and the choroidal capillaries. These pathological changes cause retinal vasopermeability increasing and the blood-retinal barrier breaking down, which in turn result in retinal hemorrhage, swelling, exudates, and retinal detachment [3].

The underlying pathophysiological mechanisms associated with hyperglycemia-induced diabetic retinopathy are complex. In the state of hyperglycemic, overproduction of reactive oxygen species (ROS) and free radicals overwhelms the intrinsic antioxidant system and reduced glutathione (GSH) levels resulting in oxidative stress and ultimately development of a pathological condition [4]. High and sustained levels of ROS cause mitochondrial DNA damage and ultimately leads to the apoptosis of RPE cells [5]. It is believed that the induction of apoptosis involves several critical steps: ROS generation, disturbance of Bcl-2 family protein balance, and reduction of mitochondrial transmembrane potential with concomitant release of mitochondrial protein cytochrome c and the subsequent activation of caspase-3 [6]. Apoptosis may also be induced via oxidative stress-mediated alterations in the mitogen activated protein kinases (MAPKs) [7]. Therefore, regulation on hyperglycemia-induced oxidative stress and associated cell death in RPE would be an important approach to protect retina from damage.

Epidemiological studies have shown that many phytochemicals found in fruits and vegetables might protect the human body against damage by ROS [8]. Therefore, phytochemicals extracted from plant sources are gaining more and more attention as potential agents for preventing and treating oxidative stress-related diseases [9]. Diosmin (diosmetin 7-*O*-rutinoside), a naturally occurring flavone glycoside readily obtained by dehydrogenation of hesperidin, is found abundantly in the pericarp of various citrus fruits [10]. This flavone improves muscular tone and vascular resistance to inflammatory processes, whose reason is that it has important pharmacological applications, being the active ingredient of certain drugs that are used in the treatment of several illnesses of the circulatory system, such as chronic venous insufficiency and rheumatic arthritis [11]. As a flavonoid, it also exhibits antihemorroidal, antioxidant and antilipid peroxidation properties and protects against the deleterious effects of free radicals [12,13,14]. Several studies have also demonstrated that diosmin has several positive effects on hyperglycemia, hypertension and hyperlipidemia [15,16,17]. Though diosmin seems to possess a multitude of biological activities to improve factors associated with diabetic complications, there is no comprehensible evidence relating to its protective role in DR.

Therefore, the aim of this study is to investigate the cytoprotective effect of diosmin against glucose exposure in a human retinal epithelial cell line as a model of DR. Taurine (2-aminoethanesulfonic acid) is the most abundant free amino acid in the retina, and is distributed mainly over the photosensory and RPE cells [18]. Experiments have proven that taurine can protect the lens from oxidative stress induced by a high concentration of glucose [19]. Thus, we used taurine as a positive control.

## 2. Results

### 2.1. Diosmin Improves the Cell Survival Rate of High Glucose Cultured ARPE-19 Cells

**Figure 1:**
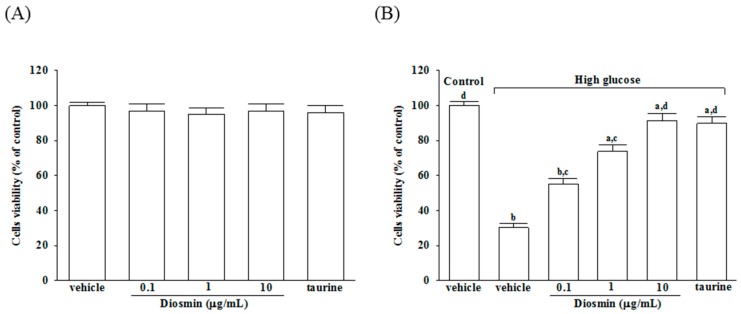
Cytotoxicity of diosmin to ARPE-19 cells. Normal glucose (**A**) or high glucose (**B**) cultured ARPE-19 cells were treated with or without different concentrations of diosmin (0.1, 1, 10 μg/mL) or taurine (1 mmol/L) for 48 h. Cell viability was conducted by MTT assay. The results are presented as the mean ± SD of five independent experiments. ^a^ *p* < 0.05 and ^b^ *p* < 0.01 compared with normal-glucose vehicle-treated group (control), respectively. ^c^ *p* < 0.05 and ^d^ *p* < 0.01 compared with high-glucose vehicle-treated group, respectively.

As shown in Figure 1A, incubation of normal glucose-treated APRE-19 cells with diosmin at 0.1, 1, 10 μg/mL or taurine (1 mmol/L) for 48 h had little or no effect on cytotoxicity (>90% viability remaining). The protective effects of diosmin against high glucose-induced cell death in ARPE-19 cells were shown in Figure 1B, that diosmin recovered the cell death caused by high glucose in a dose-dependent manner and with almost 91.2% of the cells surviving at a dose of 10 μg/mL, whereas cell viability was about 30.2% without diosmin treatment. Loss of cell viability in high-glucose culture group was similarly reversed by adding 1 mmol/L taurine (by 89.7%).

### 2.2. Diosmin Inhibits ROS Production and the Consumption of Antioxidant Biomolecules in High Glucose Cultured ARPE-19 Cells

**Figure 2:**
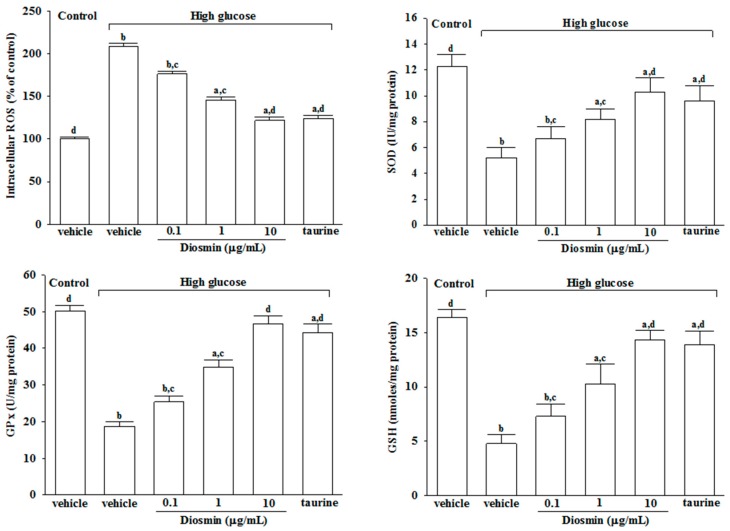
Effect of diosmin on the ROS generation and the antioxidant biomolecules in high glucose cultured ARPE-19 cells. The results are presented as the mean ± SD of five independent experiments in each column. ^a^ *p* < 0.05 and ^b^ *p* < 0.01 compared with normal-glucose vehicle-treated group (control), respectively. ^c^ *p* < 0.05 and ^d^ *p* < 0.01 compared with high-glucose vehicle-treated group, respectively.

The results showed that the mean fluorescence of intracellular ROS production was increased by about 2.3 fold in high glucose cultured cells relative to normal-glucose vehicle-treated group (Figure 2).

The increase in intracellular ROS from high glucose cultured ARPE-19 cells was decreased by treatment with diosmin in a concentration-dependent manner (Figure 2). At a diosmin concentration of 10 μg/mL, the intracellular ROS level in high glucose cultured ARPE-19 cells decreased to a near control level, which was similar to the effect produce by taurine (1 mmol/L; Figure 2). After culturing ARPE-19 cells with high glucose for 48 h, the SOD and GPx activities were found to be abundantly decreased compared to the normal-glucose vehicle-treated group (Figure 2). In high glucose cultured ARPE-19 cells, diosmin treatment resulted in a concentration-dependently increase in SOD and GPx (Figure 2). Decrease of SOD and GPx activities in ARPE-19 cells culture with high glucose were abrogated by taurine (Figure 2). The concentration of GSH in high glucose-treated ARPE-19 cells was significantly lower than those of the normal-glucose vehicle-treated group; diosmin (0.1, 1 and 10 μg/mL) and taurine (1 mmol/L) reversed the decrease of GSH (Figure 2).

### 2.3. Diosmin Prevented ARPE-19 Cells against from Apoptosis Induced by High Glucose

**Figure 3:**
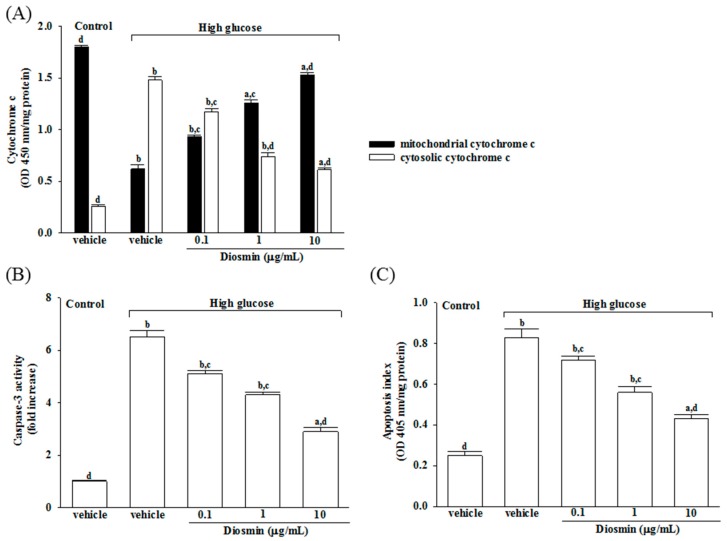
Effects of diosmin on high glucose-induced mitochondrial dysfunction in ARPE-19 cells. (**A**) Levels of cytochrome c in the cytosolic fraction or mitochondrial fractions were determined by ELISA kit; (**B**) The activity of caspase-3 in the cell lysate was measured using a colorimetric caspase-3 assay kit; (**C**) Apoptosis index was measured by detection of DNA fragmentation with the Cell death detection ELISA kit. Each column represents means ± SD (*n* = 5 per group). ^a^ *p* < 0.05 and ^b^ *p* < 0.01 compared with normal-glucose vehicle-treated group (control), respectively. ^c^ *p* < 0.05 and ^d^ *p* < 0.01 compared with high-glucose vehicle-treated group, respectively.

It has shown that decreased mitochondrial concentrations of cytochrome c were before increased cytosolic concentrations in ARPE-19 cells cultured under high glucose, and that diosmin inhibits the release of cytochrome c from mitochondria to cytoplasm in a concentration (0.1, 1, and 10 μg/mL)-dependent manner (Figure 3A).

The level of apoptic markers caspase-3 was 6.5-fold higher in high glucose cultured ARPE-19 cells compared with normal-glucose vehicle-treated group (Figure 3B). Diosmin concentration (0.1, 1 and 10 μg/mL)-dependent downregulated caspase-3 activity to 5.1-, 4.3-, and 2.9-fold, respectively, relative to the levels in their vehicle-treated counterpart group (Figure 3B).

High glucose caused a 3.2-fold increase of apoptosis rate in ARPE-19 cells and diosmin treatment attenuated this enhancements concentration (0.1, 1, and 10 μg/mL)-dependently (Figure 3C). The apoptosis rate dramatically decreased to 51.8% in high glucose cultured ARPE-19 cells with diosmin (10 μg/mL) treatment relative to the levels in their vehicle-treated counterpart gropup (Figure 3C).

### 2.4. Diosmin Regulated Protein Expression of Bax and Bcl-2 in High Glucose Cultured ARPE-19 Cells

**Figure 4:**
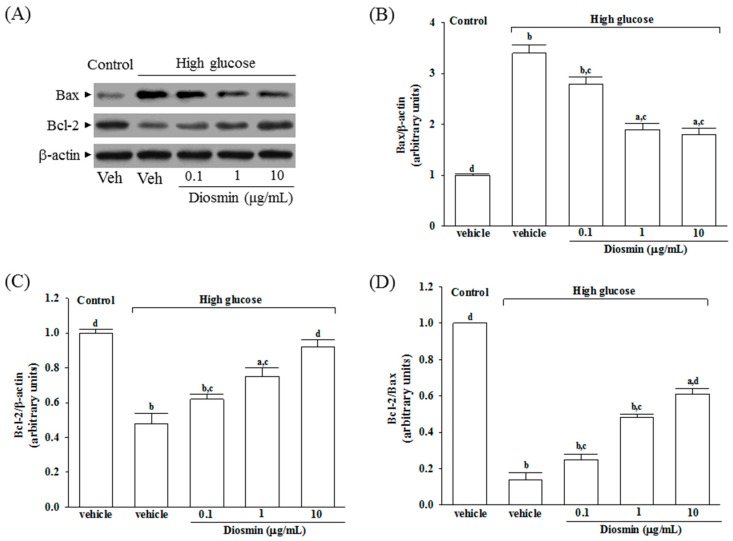
Effect of diosmin on Bax and Bcl-2 expression in high glucose cultured ARPE-19 cells. (**A**) The photographs were representatives the Western blots for Bax and Bcl-2 protein. Five western blots per group were experimented; (**B**) Relative intensities of Bax protein (fold to β-actin); (**C**) Relative intensities of Bcl-2 protein (fold to β-actin); (**D**) Ratio of the relative intensities of Bcl-2 to Bax (Bcl-2/Bax). Each column represents means ± SD (*n* = 5 per group). ^a^ *p* < 0.05 and ^b^ *p* < 0.01 compared with normal-glucose vehicle-treated group (control), respectively. ^c^ *p* < 0.05 and ^d^ *p* < 0.01 compared with high-glucose vehicle-treated group, respectively.

The protein expression of Bax was higher in the high glucose cultured ARPE-19 cells compared with the control group; diosmin attenuated the high glucose-induced expression of Bax in a concentration (0.1, 1, and 10 μg/mL)-dependent manner (Figure 4A,B). In addition, diosmin reversed the reduced amount of Bcl-2 that were detected in ARPE-19 cells cultured under high glucose (Figure 4A,C). The expression ratio of Bcl-2 to Bax was greatly decreased in high glucose cultured ARPE-19 cells than that in normal-glucose vehicle-treated group (Figure 4D). Diosmin treatments concentration (0.1, 1, and 10 μg/mL)-dependently enhanced the ratio of Bcl-2 to Bax compared to the level in their vehicle-treated counterpart group (Figure 4D).

### 2.5. Diosmin Inhibits Mitogen-Activated Protein Kinase Activation in High Glucose Cultured ARPE-19 Cells

**Figure 5:**
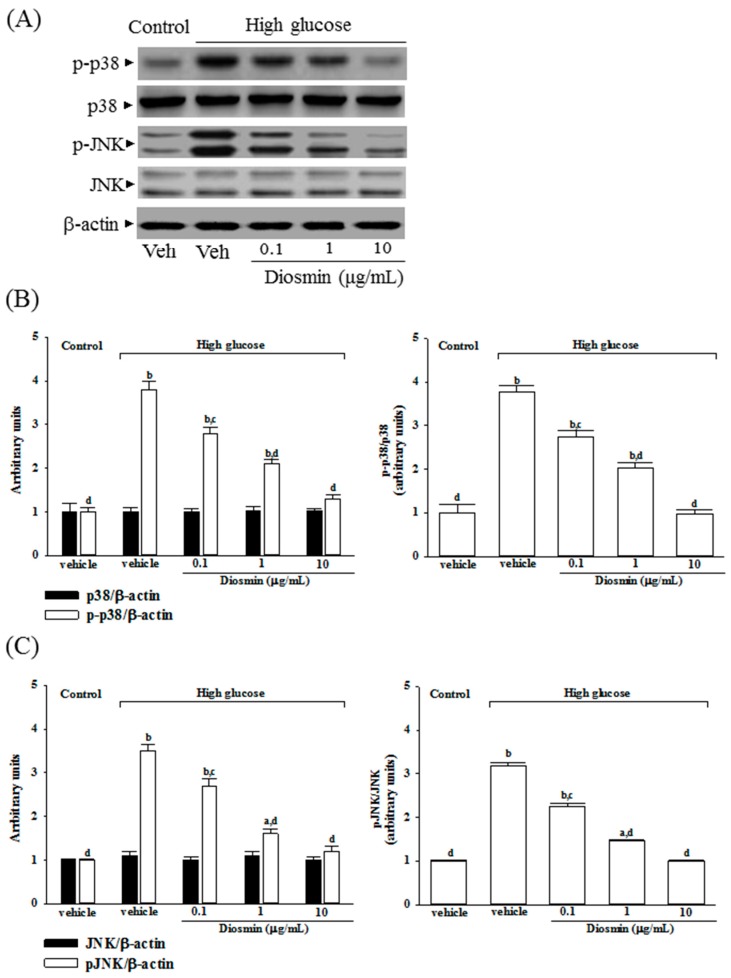
Effect of diosmin on p38 and JNK activation in high glucose cultured ARPE-19 cells. (**A**) The photographs were representatives the western blots for p-p38, p38, p-JNK, JNK and β-actin. Five western blots per group were experimented; (**B**) The relative intensities of phosphorylated and total p38 protein (fold to β-actin) were determined and the p-p38/p38 ratio was calculated; (**C**) The relative intensities of phosphorylated and total JNK protein (fold to β-actin) was determined and the p-JNK/JNK ratio was calculated. Each column represents means ± SD (*n* = 5 per group). ^a^ *p* < 0.05 and ^b^ *p* < 0.01 compared with normal-glucose vehicle-treated group (control), respectively. ^c^ *p* < 0.05 and ^d^ *p* < 0.01 compared with high-glucose vehicle-treated group, respectively.

No change was noted in the protein level of p38 and JNK in ARPE-19 cells cultured under high glucose compared with normal-glucose vehicle-treated group (Figure 5A). Diosmin did not influence the protein expression on p38 and JNK in high glucose cultured ARPE-19 cells (Figure 5A). The immunoblot results showed that the phosphorylation of p38 and JNK were 3.8- and 3.5-fold greater in the high glucose cultured ARPE-19 cells than that in normal-glucose vehicle-treated group, respectively (Figure 5B,C). High glucose-induced upregulation on p38 and JNK phosphorylation in ARPE-19 cells were significantly reversed in diosmin (10 μg/mL) treatment (56.6% decreases in p-p38 and 57.1% decreases in p-JNK, relative to those in their vehicle-treated counterpart; Figure 5B,C).

High glucose markedly elevated the ratio of p-p38/p38 and p-JNK/pJNK by 3.7- and 3.2-fold in ARPE-19 cells relative to those in their vehicle-treated counterpart, respectively (Figure 5B,C). Treatment of high glucose cultured ARPE-19 cells with diosmin (10 μg/mL) obviously downregulated the ratios of p-p38/p38 and p-JNK/JNK to 1.0- and 1.1-fold relative to those in normal-glucose vehicle-treated group (Figure 5B,C).

## 3. Discussion

The morbidity and mortality associated with diabetes arise from the myriad complications of the disease. One of the most explored hypotheses to explain the onset of complications is associated with a hyperglycemia-induced increase in oxidative stress [20]. ROS is produced by oxidative phosphorylation. Once formed, ROS deplete antioxidant defenses, rendering the affected cells and tissues more susceptible to oxidative damage [4]. Apoptosis may arise when the amount of ROS produced in the mitochondria cannot be handled by radical-scavenging cellular antioxidants [6]. Therefore, it is exceedingly important to protect cells and tissues from oxidative damage via maintaining proper activities of antioxidants. Regarded as the major enzyme against oxidative damage in the human body, SOD is a first-line defense in resisting free radicals and the superoxide negative ion [21]. GSH, a tripeptide molecule present in RPE cells, is an essential electron donor to GPx in the reduction of hydroperoxides [22]. GSH works synergistically with the other cellular antioxidants to neutralize and thereby prevent or diminish oxidative stress [23]. We demonstrated that diosmin had potent antioxidant properties in high glucose-treated ARPE cells, because the activities of SOD, GPx and its antioxidant partners GSH were significantly fortified, but the level of ROS was significantly reduced by diosmin. Thus, our results suggest that diosmin has potential to overcome the hyperglycemia-induced reactive oxygen species toxicity to RPE cells; these actions of diosmin were similar to that produced by taurine. Taurine found in the retina fights oxidative stress, especially in diabetes, and helps restore deficient levels of nerve growth factor, required for maintaining retinal health [19]. Taurine supplementation has been shown to ameliorate diabetic retinopathy [24]. As a result, it is valuable to clarify whether the protective effects of diosmin on retinal abnormalities under high glucose is linked to the amelioration of oxidative stress and relevant cell death. These findings were consistent with other studies which also reported the strong antioxidant sequestering effects of diosmin [13,14].

An excessive amount of ROS can induce mitochondrial permeability transition and the release of cytochrome c into the cytoplasm, which is an important event during cell apoptosis and necrosis [25]. Bax and Bcl-2 are putative members of the Bcl-2 family, which primarily regulate cell apoptosis [26]. It has been demonstrated that an up-regulation of Bax with a concomitant decrease in the expression of Bcl-2 changes the permeability of the mitochondrial membranes, which also results in the release of cytochrome c and the subsequent activation of caspases [27]. Caspase-3 serves as the key effector enzyme in cell death through receptor-mediated or mitochondria dependent-induced apoptosis. Our study indicated that glucose induced apoptosis was followed by an increase in Bax expression and a decrease in Bcl-2 expression, which were prevented by diosmin treatment in a dose-dependent manner. The diosmin-induced cell protection may have been mediated by normalizing the Bcl-2/Bax level. Thus, diosmin protects against RPE cells apoptosis partly by restoring the balance of anti-apoptotic and pro-apoptotic proteins which could be considerable. In addition, diosmin inhibits the release of cytochrome c from mitochondria to cytoplasm induced by high glucose; the inhibitory effect of diosmin paralleled the reduction in caspase-3 activation. Therefore, the antiapoptotic effect of diosmin on RPE cells is caspase-3 dependent. These data indicated that the mitochondrial cytochrome c-mediated caspase-3 activation pathway can be inhibited by diosmin. Therefore, diosmin could reduce high glucose-induced oxidative stress and retinal apoptosis through attenuating ROS-mediated mitochondria dysfunction.

It is known that the mitogen-activated protein kinases (MAPKs) are involved in both cell growth and death, which can be activated by ROS [28]. Among the most widely studied MAPK families are extracellular-signal regulated kinases 1 and 2 (ERK1/2), p38 MAP kinases, and c-Jun N-terminal kinases (JNKs) [29]. It is widely acknowledged that p38 and JNK MAPKs are pro-apoptotic whereas ERKs are the modulators of cell survival after reperfusion [29]. Therefore, regulating the activity of MAPK signaling pathway, particularly the activation of JNK and p38, is vital to protect cells from ROS injury and cellular death [30]. In the present study, high-glucose medium significantly promoted the apoptosis rate in ARPE-19 cells and the phosphorylation degree of p38 and JNK were significantly increased. These results are in compliance with previous studies demonstrating that high glucose induces apoptosis in ARPE-19 cells through the activation of p38 and JNK pathway [31]. Compared with the high-glucose group, the phosphorylation of p38 and JNK were both decreased in the diosmin group, suggesting that diosmin exerts its anti-apoptotic effect through the modulation of ROS-mediated p38 MAPK and JNK signaling pathway.

The RPE plays a major part in the development and functioning of the outer retina. Without RPE, the retinal photoreceptor cells, and vision itself, could not function [2]. The present study reveals the protective effect of diosmin, a natural citrus flavone of hesperidin derivative, on high glucose-induced oxidative injury in human RPE cells in vitro. By attenuating ROS-mediated mitochondria dysfunction and blockade of MAPKs phosphorylation, the molecular mechanisms underpinning the protective effect of diosmin may be mediated through the suppression of high glucose-induced oxidative stress and apoptosis. These effects of diosmin will be beneficial in the treatment of DR.

## 4. Materials and Methods

### 4.1. Cell Culture and Treatment

The human retinal pigment epithelium cell line (ARPE-19 cells) was purchased from Bioresource Collection and Research Center (BCRC 60383) of the Food Industry Research and Development Institute (Hsinchu, Taiwan). ARPE-19 cells were inoculated 2.0 × 10^4^ cells/well concentration in 24-well plates, and incubated with DMEM/F12 conditioned medium supplemented with 10% FBS and penicillin-streptomycin (100 U/mL and 100 μg/mL) at 37 °C in a 5% CO_2_ atmosphere. After incubation for 4 day with normal (5 mmol/L) concentration of D-glucose, the medium in the wells was removed and the cells exposed respectively to normal (5 mmol/L, control) and high (30 mmol/L, high glucose) concentrations of D-glucose plus diosmin (Sigma-Aldrich, St. Louis, MO, USA) (Cat. No. 61382, purity ≥ 90%) at separate concentration (0.1, 1, 10 μg/mL), or taurine (Sigma-Aldrich; Cat. No. 61382, purity ≥ 99%) at 1 mmol/L for another 48 h. Subsequently, we assessed cell viability, antioxidant enzyme activities, reactive oxygen species (ROS) generation, and several apoptotic indexes. Diosmin or taurine powder was dissolved in dimethyl sulfoxide (DMSO; Sigma-Aldrich) to create a stock at a concentration of 10 mg/mL, which was subsequently diluted in culture medium to the corresponding concentration for subsequent experiments. DMSO was used as the vehicle control in the experiments. The final DMSO concentration did not exceed 0.1% (*v*/*v*), a concentration that did not affect cell viability.

### 4.2. Cell Viability Assay

The viability of ARPE-19 cells exposed to normal or high glucose plus either diosmin or taurine was determined by a 3-(4,5-dimethylthiazol-2-yl)-2,5 diphenyl tetrazolium bromide (MTT; Sigma-Aldrich) assay, as previously described [32] with some modifications. Briefly, cells were seeded in 96-well plates a density of 3000 cells per well and incubated for 24 h. Subsequently, the cells were treated with diosmin or taurine for 48 h. At the end of the incubation, the cells in each well were washed with phosphate-buffered saline (PBS), and then, 100 μL (0.5 mg/mL) of MTT solution was added to each well. After a 4 h incubation at 37 °C, the MTT solution from each well was discarded and 100 μL of DMSO was added to each well and shaken for 5 min to solubilize the formazan formed in the viable cells. The absorbance of the samples was measured at 570 nm using a microplate reader (MTP-800; CORONA, Tokyo, Japan). Background-subtracted optical density values were normalized with the drug-free control and expressed as viability percentages. Each experiment was performed in three wells and was duplicated at least five times.

### 4.3. Detection of Intracellular ROS

Intracellular accumulation of ROS was estimated using the fluorescent dye H_2_DCFDA, which is converted to a membrane which is an impermeable and highly fluorescent compound, dichlorofluorescin diacetate (DCF), in the cell in the presence of ROS [33]. In brief, cells were seeded at a density of 2.4 × 10^5^ cells/well in 6-well plates and incubated for 24 h. After treatment with or without diosmin or taurine for 48 h, the cells were rinsed 2 times using PBS and incubated with 10 μmol/L of DCFH-DA (Sigma-Aldrich) in serum-free medium at 37 °C for 30 min according to the manufacturer’s instructions. Cells were trypsinized, washed and resuspended in PBS, and then sent for flow cytometry analysis using FACSCalibur flow cytometer (Becton-Dickinson, Franklin Lakes, NJ, USA) with CellQuestPro software (© 2002, Becton-Dickinson). The percentage of fluorescence-positive cells was measured at an excitation wavelength of 488 nm and an emission wavelength of 525 nm by a multi-detection microplate reader (SpectraMax M5, Molecular Devices, Sunnyvale, CA, USA).

### 4.4. Assay of Antioxidant Enzymes

The cells were seeded at a density of 2.4 × 10^5^ cells/well in 6-well plates and incubated for 24 h and then treated with or without diosmin or taurine for 48 h. Estimations of antioxidant enzymes such as superoxide dismutase (SOD) and glutathione peroxidase (GPx) were performed by Superoxide Dismutase (SOD) Activity Colorimetric Assay Kit (Cat. No. K335) and Glutathione Peroxidase Activity Colorimetric Assay Kit (Cat. No. K762) from Bio Vision, Inc. (San Francisco, CA, USA), following the manufacturer’s instructions, respectively. Enzyme activities (IU/mg protein) in total retinal protein extraction were measured. The level of glutathione (GSH) was estimated using Glutathione Colorimetric Assay Kit (Bio Vision, Inc., Cat. No. K621). The absorbance of each sample was read at 405 nm. The concentrations of GSH in the samples were calculated according to the standard glutathione calibration curve.

### 4.5. Measurement of Cytochrome C Release

The Mitochondrial Fractionation kit (Active Motif, Inc., Carlsbad, CA, USA, Cat. No. 40015) was used to isolate mitochondrial and cytosolic fractions from cells under the manufacturer’s instructions. Briefly, cells were seeded at a density of 2.4 × 10^5^ cells/well in 6-well plates and incubated for 24 h and then treated with or without diosmin or taurine for 48 h. The treated or untreated ARPE-19 cells were scraped and spun twice at 600× *g* for 5 min. Ice-cold 1× cytosolic buffer was added and the cell pellet was resuspended and incubated on ice for 15 min. Cells were homogenized and the lysate was spun twice at 800× *g* for 20 min. The resultant supernatant contained the cytosol, including mitochondria; the supernatant was spun at 10,000× *g* for 20 min to pellet the mitochondria. The mitochondrial pellet was washed and spun with 1× cytosolic buffer at 10,000× *g* for 10 min, and then lysed by adding Complete Mitochondria Buffer followed by incubation on ice for 15 min, the result of which was the mitochondrial fraction. At the same time, the supernatant was centrifuged at 16,000× *g* for 25 min. The centrifuged supernatant was the cytosolic fraction. The protein concentration was measured by using a Bio-Rad protein assay. After isolation of mitochondria and cytosolic fraction, the Cytochrome C ELISA kit (Abcam plc., Cambridge, MA, USA, Cat. No. ab110172) was used to measure the level of cytochrome c according to the manufacturer’s instructions.

### 4.6. Caspase-3 Activity Assay

The cells were seeded at a density of 2.4 × 10^5^ cells/well in 6-well plates and incubated for 24 h and then treated with or without diosmin or taurine for 48 h. The activity of caspase-3-like protease in the lysate was measured using a colorimetric caspase-3 assay kit (Sigma-Aldrich, Cat. No. CASP3C) according to the manufacturer’s protocol. In brief, cytosolic protein (100 μg) was mixed with caspase-3-specific substrate acetyl-Asp-Glu-Val-Asp-*p*-nitroanilide (final concentration, 200 μmol/L) and incubated at 37 °C for 90 min. The absorbance was read at 405 nm. To confirm that substrate cleavage was the result of/ had to do with caspase activity, lysates were incubated in the presence of the caspase-3-specific inhibitor acetyl-DEVD-CHO (final concentration, 20 μmol/L) at 37 °C, before the addition of substrate. The value (in arbitrary absorbance units) of the absorbance signal of the inhibited sample was subtracted from that of the non-inhibited sample.

### 4.7. Quantification of Apoptosis

The cells were seeded at a density of 2.4 × 10^5^ cells/well in 6-well plates and incubated for 24 h and then treated with or without diosmin or taurine for 48 h. Cell death detection ELISA kit (Roche Molecular Biochemicals, Mannheim, Germany, Cat. No.1544675) was used to quantitatively detect the cytosolic histone-associated DNA fragmentation under manufacturer’s instructions. Briefly, the cytoplasmic extracted from cells were used as an antigen source in a sandwich ELISA with a primary antihistone mouse monoclonal antibody coated to the microtiter plate and a second anti-DNA mouse monoclonal antibody coupled to peroxidase. The amount of peroxidase retained in the immunocomplex was determined photometrically by incubating with 2,2′-azino-di-[3-ethylbenzthiazoline sulfonate] (ABTS) as a substrate for 10 min at 20 °C. The change in color was measured at a wavelength of 405 nm by using a Dynex MRX plate reader controlled through PC software (Revelation, Dynatech Laboratories, El Paso, TX, USA). The optical density (OD) reading was then normalized to the total amount of protein in the sample and the data were reported as an apoptotic index (OD_405_/mg protein) to indicate the level of cell death.

### 4.8. Western Blot Analysis

The cells were seeded at a density of 2.4 × 10^5^ cells/well in 6-well plates and incubated for 24 h. After treatment with or without diosmin or taurine for 48 h, the cells were rinsed 2 times using PBS and collected. For western blot analysis, cells were collected and lysed in ice-cold RIPA buffer (50 mmol/L Tris-HCl, 150 mmol/L NaCl, 1 mmol/L EGTA, 1 mmol/L EDTA, 20 mmol/L NaF, 100 mmol/L Na3VO4, 1% Nonidet P-40 (NP-40), 1% Triton X-100, 1 mmol/L phenylmethylsulfonyl fluoride (PMSF), 10 mg/mL aprotinin and 10 mg/mL leupeptin) for 30 min. Protein concentration was determined by the Bradford method and samples containing 50 μg of protein were analyzed by Western blot analysis. Protein was separated by electrophoresis on 10% sodium dodecyl sulfate-polyacrylamide gel electrophoresis (SDS-PAGE) and transferred electrophoretically to polyvinylidene difluoride (PVDF) membranes. Membranes were blocked with 5% non-fat dry milk in Tris-buffered saline Tween (20 mmol/L Tris, pH 7.6, 137 mmol/L NaCl, and 0.1% Tween 20) for three hours at room temperature, followed by an overnight incubation at 4 °C with primary antibodies against Bcl-2 (Santa Cruz Biotechnology, Inc., Santa Cruz, CA, USA, Cat. No. sc-492), Bax (Santa Cruz Biotechnology, Inc., Santa Cruz, CA, USA, Cat. No. sc-526), JNK (Santa Cruz Biotechnology, Inc., Cat. No. sc-137020, p-JNK (Thr 183/Tyr 185) (Santa Cruz Biotechnology, Inc., Cat. No. sc-6254), p38 MAPK (Cell Signaling Technology, Beverly, CA, USA, Cat. No. 9212), p-p38 MAPK (Thr180/Tyr182) (Cell Signaling Technology, Cat. No. 9211), or β-actin (Santa Cruz Biotechnology, Inc., Cat. No. sc-130656). All antibodies were used at a dilution of 1:1000. After washing three times with Tris-buffered saline Tween 20 (TBST), incubation with appropriate horseradish peroxidase-conjugated secondary antibodies was performed for one hour at room temperature. After another three TBST washes, the immunoreactive bands were visualized by enhancing chemiluminescence (Amersham Biosciences, Buckinghamshire, UK) under the manufacturer’s instructions. Band densities were determined using ATTO Densitograph Software (ATTO Corporation, Tokyo, Japan) and quantified as the ratio to β-actin. The mean value for samples from the vehicle treated cells cultured under normal glucose (control) on each immunoblot, expressed in densitometry units, was adjusted to a value of 1.0. All experimental sample values were then expressed relative to this adjusted mean value. Tissue sections were sampled from five independent experiments.

### 4.9. Statistical Analysis

Data are expressed as the mean ± standard deviation (SD). Statistical analysis and graphics were performed with a SigmaPlot 12.3 program (version 2016, Systat Software Inc., San Jose, CA, USA). Statistical analysis was performed with one-way analysis of variance (ANOVA). Dunnett range post-hoc comparisons were used to determine the source of significant differences, where appropriate. A *p*-value < 0.05 was considered statistically significant.

## 5. Conclusions

Our data show that diosmin did protect human RPE cells against high glucose-induced oxidative injury in vitro through attenuating JNK and p38 signaling pathway, which further decreased the expression levels of cytochrome c, Bax and caspase-3 while increased Bcl-2 expression. The finding of this study sheds light on the pharmacological application of diosmin to prevent and offer therapy of high glucose-induced retinal oxidative damage, and provides the theoretical basis for further development of diosmin for diabetic retinal diseases.
